# Computer Vision Based Pothole Detection under Challenging Conditions

**DOI:** 10.3390/s22228878

**Published:** 2022-11-17

**Authors:** Boris Bučko, Eva Lieskovská, Katarína Zábovská, Michal Zábovský

**Affiliations:** University Science Park UNIZA, University of Žilina, Univerzitná 8215/1, 010 26 Žilina, Slovakia

**Keywords:** pothole detection, pavement distress, adverse conditions, Yolo v3

## Abstract

Road discrepancies such as potholes and road cracks are often present in our day-to-day commuting and travel. The cost of damage repairs caused by potholes has always been a concern for owners of any type of vehicle. Thus, an early detection processes can contribute to the swift response of road maintenance services and the prevention of pothole related accidents. In this paper, automatic detection of potholes is performed using the computer vision model library, You Look Only Once version 3, also known as Yolo v3. Light and weather during driving naturally affect our ability to observe road damage. Such adverse conditions also negatively influence the performance of visual object detectors. The aim of this work was to examine the effect adverse conditions have on pothole detection. The basic design of this study is therefore composed of two main parts: (1) dataset creation and data processing, and (2) dataset experiments using Yolo v3. Additionally, Sparse R-CNN was incorporated into our experiments. For this purpose, a dataset consisting of subsets of images recorded under different light and weather was developed. To the best of our knowledge, there exists no detailed analysis of pothole detection performance under adverse conditions. Despite the existence of newer libraries, Yolo v3 is still a competitive architecture that provides good results with lower hardware requirements.

## 1. Introduction

Road infrastructure and road transport are key elements of the developed economy. Roads are essential for gaining access to employment, education, health care and other services. The issue of the attractiveness of driving a passenger vehicle for normal trip purposes is described in several traffic models and studies [1,2]. Yet, insufficient road maintenance often occurs and many cities and roads around the world suffer from pothole issues. While some of these problems are minor and have little impact on traffic safety and consistency, others can lead to dangerous situations. The growing number of road accidents leading to vehicle damage and serious injuries necessarily requires better management of these issues. 

A pothole can be characterized as damage of road surface. Usually, it is a hole structure which has developed over a specific time period as a result of traffic and weather. Potholes can cause damage to car wheels (dents in the rims) and suspension parts, deflated tires, a wheel alignment problem, damage to the undercarriage, and so on [3]. According to the Fact Sheet of Pothole Damage, drivers of the United States have spent USD 15 billion to repair vehicle damages caused by potholes between 2012 and 2016 [4]. Worn out technical conditions of roads is also considered an aspect that lowers the resilience of transport [5]. Keeping the road infrastructure safe from potholes and tracking the condition of newly discovered and existing potholes is a challenging task. It is necessary, not only for the traffic safety and road administration, but also for autonomous vehicle driving and navigation services where a major challenge is posed by a complex urban environment [6]. If the location of road damage could be automatically detected and shared with other vehicles as well as with road maintenance services, such a system would contribute to improved road safety [7].

Pothole detection and monitoring is a current topic across Europe, but still no unified or standardized solution has been provided. There are various approaches for pothole detection which unfortunately do not ensure accuracy in challenging light and weather conditions. Therefore, an extensive dataset was developed and tested within chosen models, which could serve as a starting point for further research in the future.

### The Importance of Pothole Detection

The first question that may come to the reader’s mind might be: “Is there anything we can do to reduce pothole creation?” Pothole creation is very complex process connected to weather conditions and the construction materials used to build roads. That is why potholes are a worldwide problem which does not have a straightforward solution. According to [8], there are several stages during the forming of pothole:

- Potholes begin to form when water flows into cracks and small holes in the road. These cracks and small holes are created due to road wear over time.
- The second stage is characterized by a change in temperature. When the temperature drops below freezing, the water freezes to ice and expands its volume. As a result, the road changes its shape and can rise.
- In the third stage, the road temperature rises during the day, the ice melts and the vehicles gradually disrupt the damaged road surface as they pass through.

Although most of the potholes are developed within the cold climate, the road infrastructure in warm climate areas must also deal with potholes. High temperatures damage the road surface and cause cracks and holes which transform into potholes over time.

**Figure 1:**
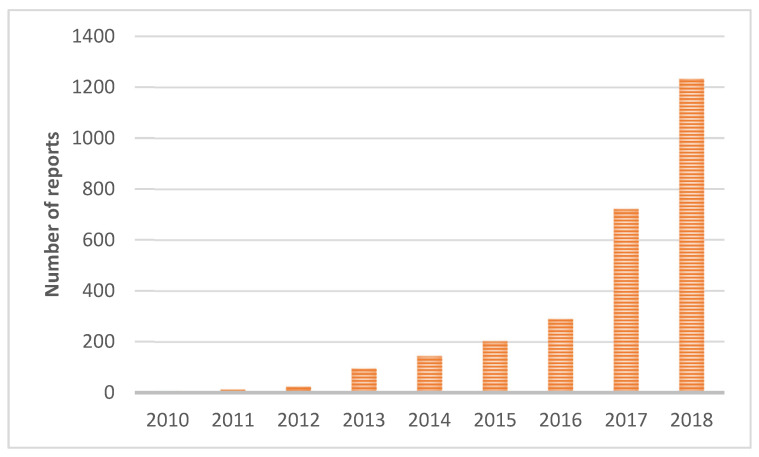
Number of pothole reports per year according to [9].

According to [9], pothole issues are one of the most discussed topics in municipalities. The number of citizen pothole reports and reports of unsatisfactory condition of road infrastructure have risen every year since 2010 in Slovakia. In 2018, the share of pothole related reports was more than 10% of the all-citizen reports across Slovakia (Figure 1).

Pothole recognition can be a difficult task for humans, let alone for machines, especially in adverse weather conditions. Among the well-known hazardous driving conditions are rain, snow, and fog. On rainy days, potholes may be hidden under puddles or may resemble puddles. Water on the car windshield can obscure the field of view and prevent the detection of road damage. Poor visibility caused by fog can easily result in pothole damage to vehicles.

The automatic detection of objects based on deep computer vision models can, to some extent, suppress various adverse influences. The common approach to improve generalization of deep neural models is to use a training dataset consisting not only of instances of road damage captured under clear weather, but also instances distorted from illumination (different daylight) and various kinds of weather conditions as presented in [10,11,12]. However, these studies did not dive deeply into adverse conditions. 

Several works deal with automatic pothole detection [10,11,13,14,15,16], where either high accuracy or real time inference was considered. Many of the authors and related works dealt with computer vision-based object detection. Although many interesting results were achieved, these studies lack consideration of various light and weather conditions. That is why we consider our work notable in terms of further research, not only for computer vision but also for intelligent transport systems, automated vehicles, and pavement condition assessment monitoring. 

We faced various challenges during our research. Data collection took place in a live transport infrastructure over a long period of time. Data annotation and post processing had to be carefully performed for proper use of the computer vision model. To ensure as much accuracy as possible, the annotation was done manually by skilled drivers and experts in the field of transportation systems and road construction. This process was laborious and labelling in bad light and weather conditions was difficult, where it was often problematic to recognize potholes and manhole covers.

Although the light conditions and adverse/hazardous weather are the main factors influencing visual data obtained by camera, detailed academic results considering weather and light conditions and even region are still missing. That is why it is important to continually develop not only new datasets for AI but also to test these datasets using various models and to define their weaknesses and strengths. Although the recent academic research studies lack mentioned attributes, these studies set up important milestones for our research. 

It is quite a challenging task to detect potholes under adverse conditions and achieve a high level of precision. The main approach to address this issue is a systematic approach which could be established by creating a detailed adverse conditions pothole dataset. The next step would be to use the created dataset in the computer vision models. This is the approach we are presenting in our paper.

In this work, a performance comparison of automatic pothole detection under degraded light or bad weather conditions is presented. This task is closely related to the development of a dataset consisting of samples collected under the adverse conditions, which is one of the contributions of this work. Another contribution is proving that older Yolo v3 is still a competitive solution mainly for lower performance hardware. 

## 2. Related Works

There are various approaches to detect road potholes—from the basic ones which involve manual in-person pothole recording to the sophisticated ones involving progressive technologies like laser scanners, recording devices and object detection.

### 2.1. Sensors and 3D Reconstruction Techniques

Road condition assessment is a very laborious task, and the cost of the specialized devices needed can often be high. Therefore, low cost solutions are sought, such as the use of mobile sensors (accelerometer or vibration sensor [17,18], magnetometer, GPS) and the concept of crowdsourcing or participatory sensing [19,20,21]. Accelerometer based approaches can be of high accuracy and are not dependent on visibility conditions. On the other hand, they can have low response time and driving through a pothole is required to perform detection [22].

The point cloud models of road discrepancies can be modelled with laser or stereo vision techniques. A 3D road profile can also be obtained using terrestrial laser scanning, but it is necessary to plan ahead, especially for maximum efficiency, where it is important to plan the correct placement of reference points, estimate the number of scanning positions and plan their correct placement to achieve the expected results [23]. LiDAR can detect objects in low visibility conditions, thus enabling the detection of potholes at night.

A stereo vision-based system can retrieve information about the size and depth (volume), as well as the position of the potholes from two simultaneously captured images [24]. Despite considerable accuracy, pothole detection based on stereo vision might have disadvantages such as computational complexity and sensitivity to motion and undesirable vibrations [10,25].

### 2.2. Two-Dimensional Vision-Based Techniques

Two-dimensional vision-based techniques consist of a variety of image processing methods, edge detection strategies such as thresholding of transitions in colors, and machine learning. K-means clustering, and subsequent black color thresholding was applied for pothole detection in [26]. Nienaber et al. [27] proposed simple image processing techniques using Canny edge detection on road surface areas. Apart from pothole edge detection, several undesirable edge instances such as shadows of leaves or edges from other vehicles were also detected. 

The pothole detection with traditional image processing approach highly depends on illumination and weather conditions. Thus, the modification of image processing techniques may be required for distinct road conditions. Moreover, this approach often consists of complicated processes requiring expertise in image processing.

Nowadays, the computer vision based deep learning algorithms present a clean and effective solution to 2D vision-based pothole recognition. When the training dataset contains a variety of samples and proper neural network architecture is chosen, deep learning algorithms can be tuned, to some extent, to handle the adverse road conditions.

The methods of visual based object detection are commonly divided into two groups: two- and one-stage object detectors [10,28]. As the names imply, two-stage detectors are composed of two separate networks: region proposal network (RPN) and object detection network. The most common example of a two-stage detection approach is a region with CNN features (R-CNN) [29]. R-CNN extracts category independent region proposals (bounding boxes) for the input image with RPN firstly, and then the feature extraction and classification (CNN + SVM) is performed on proposed regions. The drawback of R-CNN is demanding optimization and slow performance for real-time use. Faster R-CNN [30], the refinement of R-CNN architecture, further improves the inference time. 

The demand for an end-to-end system with low computing costs for real-time applications was met with the invention of one-stage object detectors. This group of detectors includes models such as Single Shot Detector (SSD) [31] and Yolo, which do not implement the region proposal step and provide direct prediction of bounding boxes and classification score for detected objects. Yolo (You Only Look Once), first introduced in 2015 [32], is one of the most prevalent object detector architectures belonging to the group of convolutional neural networks (CNN). Yolo version 1 consists of 24 convolutional layers as well as four max pooling and two fully connected layers. Yolo is prone to worse detection accuracy compared to Fast R-CNN. Moreover, small objects and objects located in clusters are difficult for Yolo to detect [28]. There have been continual improvements of the original Yolo. In 2016, Yolo v2 replaced its feature extractor with darknet-19 [33]. In 2017, Yolo v3 used the darknet-53 backbone [34]. Among the improvements on the original version are enhancement of feature extraction module, operation of upscaling and detection at different scales [28]. In 2020, Yolo v4 was proposed by Alexey Bochkovskiy in [35]. The feature extraction module of Yolo v4 is Cross Stage Partial connections based Darknet53. A novel Mosaic and Self-Adversarial Training data augmentation was proposed. Yolo v5 [36] utilizes the cross-stage partial bottleneck–based ResNet101 for feature extraction. Model types of different sizes are available: XS, S, M, and L.

Depending on the purpose of the application, it is possible to choose between higher detection accuracy achievable with a two-stage detector or high inference speed feasible with a one-stage detector. Ideally, the goal of road damage detection is to reduce the computational cost for real-time deployment and improve the accuracy. In the next section, the recent pothole detection systems based on deep neural networks are described.

Pena-Caballero et al. [11] evaluated various object detection and semantic segmentation algorithms in terms of detection speed and system accuracy. Segmentation algorithms often provide precise results but at the cost of higher computational complexity. According to the results, Yolo v3 outperformed Yolo v2 in both mAP and speed. 

Chen et al. [13] proposed a two part location-aware convolutional neural network. The localization part proposes numerous regions containing potholes and selects the most relevant regions. Then the classification network performs the recognition of potholes on selected regions.

Park et al. [14] applied three different architectures for detection of potholes: Yolo v4, Yolo v4 Tiny, and Yolo v5s. The database of 665 pothole images from Kaggle [37] was used for both training and inference. Yolo v4 and Yolo v4 Tiny achieved comparable accuracy of 77.7%, 78.7% (AP@0.5) and slightly outperformed Yolo v5s.

Ahmed [10] utilized and compared several object detection architectures, namely: Yolo v5 models (for three different model sizes) with ResNet101 backbone, YoloR and Faster R-CNN with ResNet50, VGG16, MobileNetV2, InceptionV3 backbones. Moreover, the author proposed a modified VGG16 (MVGG16) which led to successful reduction of the computational cost while maintaining detection accuracy. According to the final comparison, Faster R-CNN with ResNet50 achieved the highest precision of 91.9% with an inference time of 0.098 s for larger images. Yolo v5 had the best inference speed with inference time up to 0.009 s (Ys), but at the cost of reduced accuracy. Ys model is therefore more suitable for real-time implementation.

A summary of recent one-class (pothole) road damage detection systems is provided as Table 1. Inference speed in FPS for the given image resolution are listed. Note that the results are not directly comparable because each work uses a different dataset for training and inference.

Several road damage conditions, such as patches, cracks, bumps, and potholes, were detected with Yolo v3 in [39]. The authors used Kalman filter tracking to further improve the system accuracy. To deal with variable distances of potholes from the recording device, two lenses with different fields of view (30° and 70°) were used for the front car view capturing. The results obtained on Taiwan pavement defect image dataset (TPDID) show that the average detection accuracy reached 71% with miss rate of 29%. The compressed Yolo v3 model was used in an embedded system for pavement defect detection with the reported execution speed of 27.8 FPS with the original system accuracy.

Du & Jiao [15] improved results of Yolo v5S by incorporating enhanced feature extraction with Bidirectional Feature Pyramid Network. Further improvement was achieved by using Varifocal Loss for effective handling of the sample imbalance. This model is denoted as BV-Yolo v5S.

A summary of recent multi-class road damage detection systems is provided as Table 2. Yolo is predominant architecture achieving real-time inference speed (>30 FPS). Note that the results are not directly comparable because different datasets for training and inference were used.

### 2.3. Road Damage Datasets

Several collections of road damage data were accumulated throughout the years of research. In Table 3, some known image datasets are listed. The visual data were collected using a smartphone on a vehicle’s dashboard [11,12,38,40] or black-box/drive cameras [27,41]. Samples can also be obtained through web search [42]. Data can be collected from the vehicle view, or it can be captured right above the road discrepancy. The first type of data is considered more suitable for real-world pothole detection.

Various types of road damage such as alligator and longitudinal cracks, manhole covers, potholes and even line blur can be considered during database development. Table 4 provides the summary of common road damage categories per dataset. 

During the real-time deployment of application for data collection, a constant stream of images may result in recording of duplicate road damage. An effective way to deal with duplicate instances is to take pictures at 1 s intervals at an average car speed of 40 km/h or 10 m/s [38,40].

## 3. Materials and Methods

Light and weather conditions during the collection of road damage samples naturally affects the accuracy of object detectors. Robust datasets containing various kinds of adverse conditions combined with deep learning are often used to deal with the quality degradation of the investigated road discrepancies [10,11,12]. Lin et al. [39] performed a test on Yolo v3 under adverse conditions such the snow and rain. Their findings demonstrated that the proposed system could effectively detect potholes in difficult situations, however, none of the results of detection accuracy were presented. 

There is no detailed study on effects of adverse conditions on road damage detection using computer vision algorithms. We attempted to address this problem by developing the pothole detection dataset containing samples collected under various difficult light or weather conditions. 

### 3.1. Dataset Development

A specific road section with road damage in the industrial part of the city was selected for collection of samples. Image samples were captured on the same road segment on different dates (in May, June, July) thus the complexity of vehicle surrounding was changing throughout the days (pedestrians, passing or parked vehicles). The resolution of all images is 1920 × 1080. The labelling of main and the largest dataset that consists of clear weather images is structured as follows:

Vid _ (day_ID) _ (direction) _ (frame_ID)

where the meaning of the individual parts of the label is:

- Vid—video frames were extracted and saved to images.
- day_ID—videos were captured on different days.
- direction—data collection was performed in both directions that are marked as Ca and Pr. The designation Ca represents images recorded in the forward direction, and the designation Pr represents images recorded in the opposite direction. The abbreviations are based on the naming of local areas.
- frame_ID—video frame identifier.

Additionally, every image contains information about date and precise time of data collection. The round nature of manhole covers may be somewhat similar to the shape of potholes and therefore the misclassification by an automatic algorithm can occur. With the strong presence of potholes and manhole covers in collected images, we decided to include the manhole cover class for better generalization of computer vision model. 

A total of 1052 images under clear weather conditions were collected and annotated. Dataset further consists of four subsets of adverse conditions—Rain, Sunset, Evening and Night. The dataset statistics such as number of instances per subset and number of instances per two categories are listed in Table 5. 

Potholes occur naturally in different shapes and sizes. The smallest size of pothole that would be still relevant for a computer vision system is not precisely defined. There are cases such as the smallest road discrepancies visible by human eye that can be considered as potholes. However, a computer vision model that learned such representations effectively could produce a false classification and mistake the pothole for a small shadow or dark spot on the road. Therefore, the pothole labelling procedure is complex task, and it can be viewed as a distance dependent problem, where only the potholes that fall into some predefined threshold distance are considered. The developed dataset is publicly available at [46].

### 3.2. Yolo v3

Yolo is one of the most popular one-stage object detectors that performs in real time [34]. Yolo v3 provided accurate results and fast inference time in previous works, which dealt with the task of pothole detection [11,39]. Therefore, it was also selected for the experiments with adverse conditions in this work.

**Figure 2:**
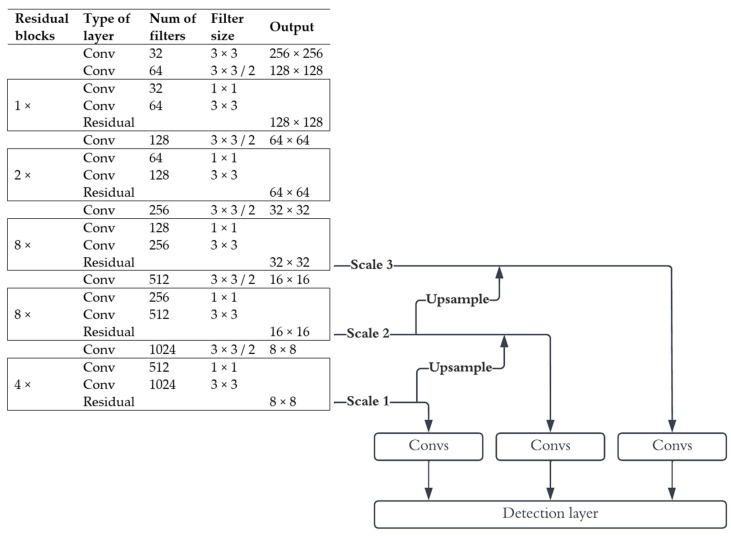
The Yolo v3 architecture.

Yolo takes the whole image as input and divides it into a grid of N×N size. Yolo v3 then uses darknet-53 backbone for feature extraction [34]. Darknet-53, as the name implies, consists of 53 convolutional layers, some of which are formed into residual blocks. Figure 2 shows the internal layout of darknet based Yolo v3 feature extraction module. This module is then followed by additional convolutional layers and operation of up sampling of feature maps and concatenation. Ultimately, Yolo v3 can predict objects on three different scales thus providing detection of large, medium, and small objects. It predicts four bounding box coordinates: t_x_ and t_y_ (center of the box), t_w_ and t_h_ (width and height). Each box is assigned a value of objectness score and the independent logistic classifiers for multilabel class predictions are also applied. Yolo implements non-maximum suppression algorithm that selectively filters out redundant bounding boxes. K-mean clustering algorithm can be applied to automatically determine the best anchor boxes for a given dataset. SPP block (spatial pyramid pooling) is often incorporated to improve the detection accuracy. SPP consists of max pooling layers at different scales, and it is used for extraction of multi scale local region features on the same input [47].

### 3.3. Evaluation Metrics

There are several criteria for assessing the accuracy of object detectors. The most used metrics are precision, recall, and average precision (AP) or mean average precision (mAP). Additionally, frame rate is an important indicator of speed of object detector.

Precision is the ratio of correctly detected instances, denoted as True Positives (TP), to all positively detected instances (TP + False positives (FP) represented in Equation (1)). Recall is the ratio of correctly detected instances to all tested instances (TP + False negatives (FN) represented in Equation (2)). The Intersection Over Union (IOU) determines the overlap between predicted and ground truth bounding boxes (Equation (3)), B_P_ and B_GT_, respectively. TP detection is then determined as a match between bounding boxes, which is above a certain threshold. FP occurs when detection falls below the threshold. FN instance denotes that the correct detection was missed. If a more than one detection is predicted for a ground truth object, a condition is defined such that only the prediction with the highest IOU is true.

(1) $$\mathrm{precision}=\frac{\mathrm{TP}}{\mathrm{TP}+\mathrm{FP}}.\;$$

(2) $$\mathrm{recall}=\frac{\mathrm{TP}}{\mathrm{TP}+\mathrm{FN}}.$$

(3) $$\mathrm{IOU}=\frac{{\mathrm{area}\;(B}_{P}\;\cap {\;B}_{\mathrm{GT}})}{{\mathrm{area}\;(B}_{P}\;\cup {\;B}_{\mathrm{GT}})}.\;$$

AP is based on computation of precision-recall or PR curve and the area under the curve (AUC). PR curve represents a trade-off between precision and recall at various thresholds. AP measure defined for The PASCAL Visual Object Classes Challenge 2010 [48] is determined from the PR curve by interpolating the precision at eleven recall levels (0,0.1,…,1) (Equation (4)). The precision at each recall level is denoted by ρ_interp_(r) and it is interpolated by taking the maximum precision measured for which the corresponding recall exceeds r. AP metric is intended for unified evaluation of both classification and detection.

(4) $$\mathrm{AP}=\frac{1}{11}\;\sum_{r\in \left\{0,0.1,\;\ldots ,1\right\}}\rho_{\mathrm{interp}}(r)\;.$$

The overall precision is indicated by mAP and it is computed as average across all *n* categories. mAP@0.5 denotes that the metric is evaluated for IoU detection threshold of 0.5. The metric mAP@[0.5:0.95], firstly used in MS COCO challenge [49], is averaged over several IoU thresholds from 0.5 to 0.95 with step of 0.05.

$$\mathrm{mAP}=\frac{1}{n}\;\overset{n}{\underset{i=1}{\sum }}{\mathrm{AP}}_{i}$$

## 4. Results

For the experiments, Ultralytic Yolo v3 model was utilized [50]. It provides several training options such as pretrained weights and three different models to test:

- Yolo v3 Tiny,
- Yolo v3,
- Yolo v3-SPP.

Since the current pothole detection task in the “wild” is focused on precision of results, Yolo v3 Tiny (designed to achieve faster inference time) was omitted from our experiments. Ultralytic implementation allows for rectangular training and provides for a wide range of augmentation techniques. An automated batch size was selected with autobatch property. Although the maximum number of epochs was selected to be 1000, the early stopping criterion enabled the model to stop training if validation metrics did not improve over time. 

The performance of Yolo v3 in terms of detection accuracy is then compared with the Sparse R-CNN model [51]. According to [51], Sparse R-CNN model provides accuracy, run-time, and training convergence performance competitive with the state-of-the-art object detectors on the large-scale COCO dataset. Our experiments are enriched with pretrained Sparse R-CNN model which was utilized from MMdetection repository [52]. More specifically, configuration file consists of ResNet-50 Feature Pyramid Network, 3× training schedule and predefined augmentation techniques (random crop and multi-scale from training images).

### 4.1. Yolo v3

**Figure 3:**
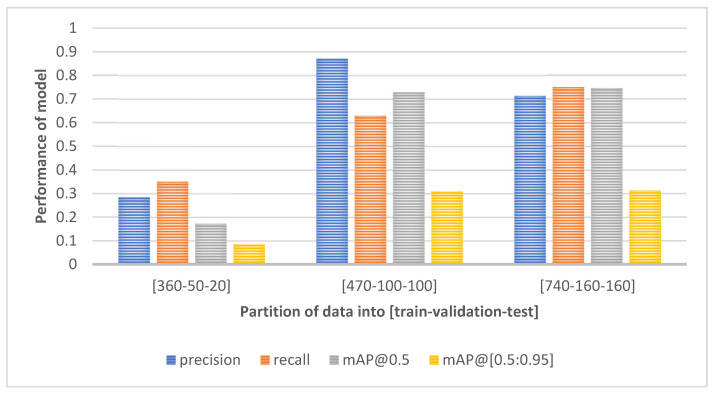
The performance of Yolo v3 under clear weather conditions during the dataset development.

Preliminary results of Yolo v3 performance on an incomplete dataset (of clear weather samples only) that was still under development are shown in Figure 3. The initial set of data was divided into training, validation, and test partitions, which are listed below the graph. The third and final subset of about 1050 “clear” images was divided according to 70-15-15% ratio. This subset was also used for training in further experiments. Naturally, the increase of image samples resulted in better model performance. Higher precision signifies a lower amount of false positive detections, whereas higher recall relates to a low false negative rate. Although the best precision was indicated in the middle partition, the final subset achieved balanced precision-recall measures as well as slightly higher mean precision metrics. 

The results of two-class recognition with the final set of images under clear weather conditions are shown in Table 6. The effects of pretrained weights, augmentation, an increase in the image size and SPP pooling module were examined. There is clear improvement of detection accuracy with the use of pretrained weights and augmentation. Naturally, the longer image size length produced better results. The highest detection accuracy was achieved using Yolo v3 with SPP module. The training time ranges from 2 h (basic Yolo v3 model without pretrained weights and augmentation) to 13 h (with SPP module and image size of 1080). The mean inference speed of Yolo v3 was ~35 ms and ~82 ms for the input image size of 640 and 1080, respectively. The use of SPP module results in slightly higher inference time. 

The effects of adverse light and weather conditions on Yolo v3 detection accuracy are given in Table 7. The natural degradation of results was observed with worsened light conditions. Both mAP@0.5 and mAP@[0.5:0.95] accuracy metrics steadily decreased from sunset and evening hours into the night hours. The detection accuracy on rainy days was also influenced by lowered light. Moreover, the rain spots on a car’s windshield contributed significantly to the increase in false detections. The real challenge for this computer vision model was low visibility at night, where it performed the worst.

After a thorough inspection of the detection outcomes, several phenomena were noticed. Reflection of the objects from the wet road or rain spots on a car’s windshield were often falsely misclassified as potholes. Small cracks and dark spots on the road might be identified as potholes in the images with low visibility. False detections were no exception even in the case of images recorded under clear weather, for instance the reflection from a car hood may be detected as a pothole. 

It is obvious that manhole covers are more easily recognized compared to potholes. The non-uniform appearance of road discrepancies naturally poses a challenge for computer vision models. Moreover, the investigated objects are small compared to the overall image of the scene, so the resolution of input image is also a significant factor for detection accuracy. Image size plays an important role, especially when driving with reduced visibility at night.

**Figure 4:**
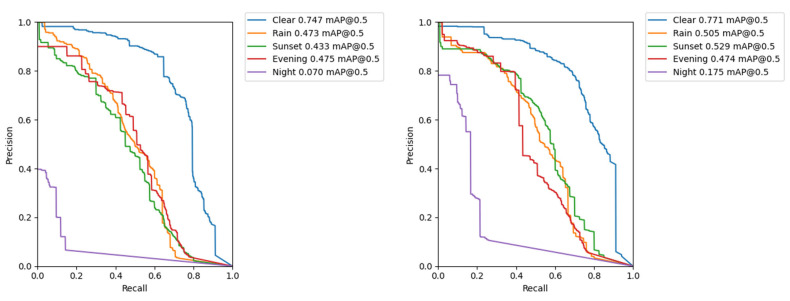
The PR curves for different conditions. (**Left**) Yolo v3 model with input image size of 640; (**Right**) Yolo v3 model with input image size of 1080.

The comparison of PR curves (see Section 3.3 above) for the subsets of different conditions is shown in Figure 4. The larger the area under the curve, the better the performance of the model in terms of detection accuracy. 

**Figure 5:**
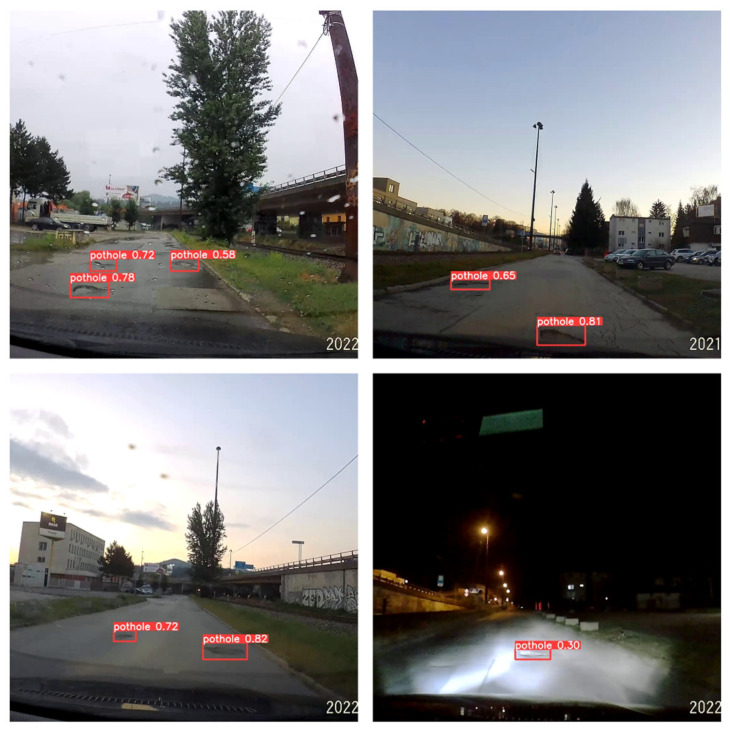
Pothole detection results in adverse conditions.

The model performance can be to some extent considered similar on a rainy day (July, 11:45 am), at sunset in winter (December, 15:45 pm) and in the summer evening (June, 9 pm). The worse detection accuracy of the computer vision model was observed in the nighttime environment. In this case, an improvement in results was noted when using larger images (input size of 1080) which could contain finer details of potholes and manhole covers. Different light and weather conditions are shown in Figure 5. The reader can clearly see how the visibility of potholes varies according to light and weather change.

### 4.2. Yolo v3-SPP

**Figure 6:**
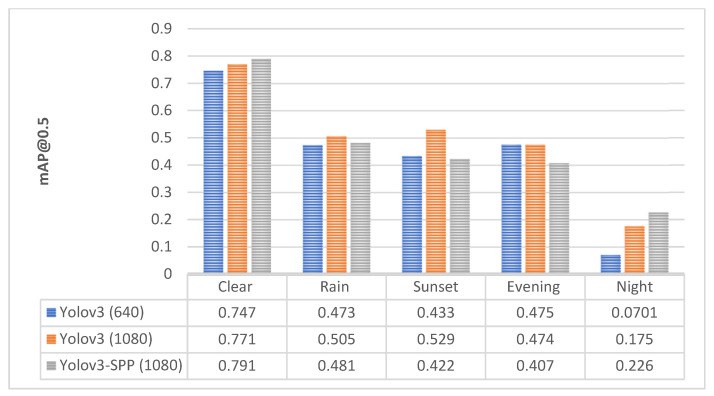
The detection accuracy of Yolo v3 for different settings.

The effects of adverse light and weather conditions on detection accuracy of Yolo v3 model with different settings (input image size of 640 and 1080, utilization of SPP) is shown in Figure 6. As mentioned previously, the larger input image size yields better detection accuracy. As can be seen in Figure 6, the input image resolution has a positive effect on the most challenging road conditions at night. Interestingly enough, Yolo v3 benefitted from SPP module mainly for clear day and night conditions; however, Yolo v3-SPP performed poorly under rainy, sunset and evening conditions. In view of this kind of deeper analysis, the suitable methods for pothole detection can be chosen based on preference of detector use case—e.g., whether the main operational time would be day or night hours.

**Figure 7:**
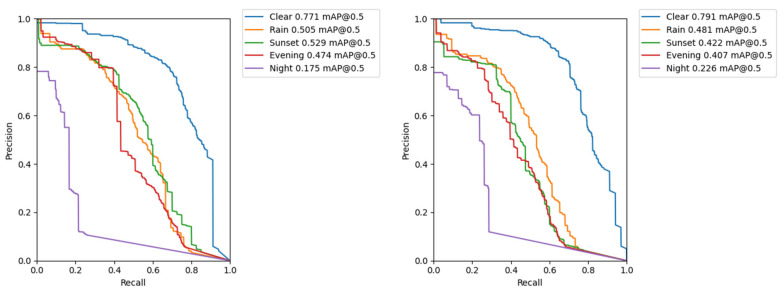
The PR curves for different conditions. (**Left**) Yolo v3 model with input image size of 1080; (**Right**) Yolo v3-SPP model with input image size of 1080.

For completeness, the comparison of PR curves of Yolo v3 (1080) and Yolo v3 (1080) with SPP module is shown in Figure 7. It can be repeatedly seen that SPP module improves detection accuracy for the worst-case detection scenario occurring at night.

### 4.3. Sparse R-CNN

The Yolo v3 is further compared with Sparse R-CNN model [51] which is, as the name implies, completely sparse, i.e., object recognition head is given a fixed sparse collection of learnt object proposals. This fully sparse model is different from the one-stage detectors (Yolo) which are based on proposal of dense candidates and two-stage detectors (R-CNN or Faster R-CNN) which offer sparse set of foreground proposals from dense region candidates obtained through region proposal techniques. Within the Sparce R-CNN, the total number of object candidates is reduced significantly and non-maximum suppression step for reduction of redundant proposals is not utilized. Sparce R-CNN provides for accuracy and run-time performance competitive with the state-of-the-art object detectors on the COCO dataset.

Table 8 shows the comparison of detection accuracy of Yolo v3/SPP and Sparse R-CNN. In our case, the performance of both architectures under the clear dataset can be considered similar. In terms of model size, Yolo v3 is up to three times smaller than Sparse R-CNN.

**Figure 8:**
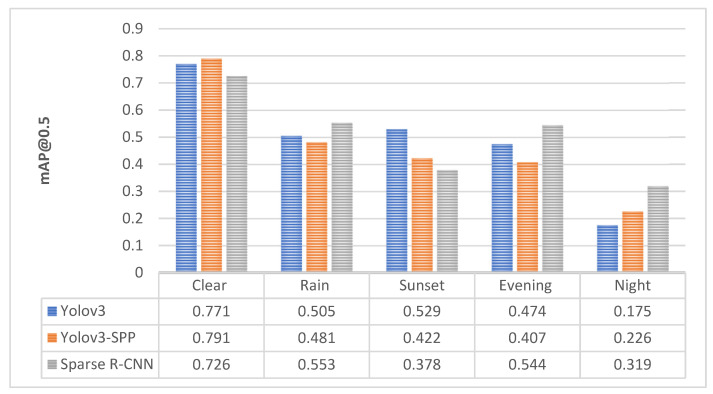
The comparison of detection accuracy of Yolo v3, Yolo v3-SPP and Sparse R-CNN.

The detection accuracy of Sparse R-CNN under different light and weather conditions is shown in Figure 8. Sparse R-CNN outperformed Yolo v3 in almost all adverse conditions. It was mainly beneficial for low light scenarios in evening and at night. Yolo v3 worked better under the clear weather condition and sunset in winter. Sunset dataset can be considered of brighter light conditions than summer evening and night. Each model deals with degraded conditions differently. Therefore, such a detailed analysis is suitable for object detectors performance comparison. 

## 5. Discussion

According to related works and studies described in Section 2.3, comparative analysis is not a straightforward task. This is due to the fact that each work utilized a different dataset. For instance, experiments with data captured right above the road discrepancy tend to achieve higher detection results than experiments with data collected from the vehicle’s perspective. Moreover, different hardware environments are usually employed for conducting experiments. 

In general, similar papers do not consider pothole detection under adverse conditions. Although we have presented some, they do not provide such level of adverse condition details as was proposed in our dataset and models. The results we showed in our paper have not been presented in this form before, even in the related literature. We see this as a positive development, as it is also an opportunity for other scientists to enrich the field with their research results. 

In this paper, comparable accuracy to other works was achieved using Yolo v3-SPP (1080 × 1080) with mAP@0.5 up to 0.791 in multi-class object detection (potholes and manhole covers). Although higher accuracy was at the expense of reduced detection speed, the near real time inference was achieved using Yolo v3 with input image size of 640 × 640 and mAP@0.5 of 0.747. 

A detailed examination of the detection results under adverse conditions revealed various oddities. In images with limited visibility, little cracks or dark areas on the road may be mistaken for potholes. On rainy images, reflection of the objects from the wet road or rain spots on the car’s windshield can also be falsely misclassified as potholes. The detection accuracy of Yolo v3 model on a rainy day, at sunset and in the evening can be considered similar to some extent.

Very low visibility at night, posed the major challenge for Yolo v3. Detection accuracy for Yolo v3 (640 × 640) in terms of mAP@0.5 dropped from 0.747 to 0.0701, which represents close to 90% decrease in accuracy. In this case, an improvement in results was observed when using larger input size of images that could contain finer details of potholes and manhole covers. Further improvement was achieved with utilization of SPP module that is used for extraction of multiscale local region features. However, not all cases were improved with SPP solution. We were able to improve the pothole object detection in low light conditions using Sparse R-CNN. As was illustrated in the Figure 8, the major improvement was recognized at night object detection, where mAP@0.5 rose from 0.226 (Yolo v3-SPP) to 0.319 (Sparse R-CNN). If the effects of adverse conditions on detection accuracy can be measured, it is possible to take the precautions to deal with them more effectively. 

Even though we achieved relatively high accuracy, there is an assumption that the results could be further improved, and we perceive this as one defect of the method used. This defect can be solved by upgrading the method and switching to Yolo 4 or Yolo 5. Nevertheless, it is good to emphasize that the method using the Yolo 3 library significantly reduces hardware requirements and processing time, which was also the reason why we decided to use this method. Our continuous research will focus also on comparison of newer Yolo versions with the results presented in this paper.

## 6. Conclusions

Encounters with road damage such as potholes or road cracks are almost unavoidable when traveling. Safety, comfort, and avoidance of damage to vehicle during driving are of great importance to road users. Nowadays, deep learning algorithms present an effective solution to pothole recognition. In this paper, Yolo v3 the computer vision model is used to automatically detect potholes. Because visual object detection performance is negatively affected by various circumstances such as reduced light or bad weather, the detection of potholes under adverse conditions was proposed. For this purpose, a dataset that consists of image collections of diverse light and weather conditions was developed.

The results using Yolo v3 clearly showed a fairly high success rate. From the results, we can conclude that Yolo v3 is still a suitable alternative for pothole detection when it is possible to achieve results with lower computing time and significantly lower hardware requirements. Although using Sparse R-CNN brought better results in low light conditions, Yolo v3 proved better performance under brighter light conditions.

The main benefit of our work is the provision of an elementary foundation for further research. Our research provides a detailed dataset and uses established methodology for computer vision-based object detection. We have proven that pothole recognition under adverse conditions is quite a novelty topic with a few research results so far, which opens many possibilities for continued research.

In future experiments, the update of road damage dataset to other types of adverse conditions will be proposed as well as further tests with various object detectors and other relevant libraries [53]. We will also focus on running models with different hardware configurations with the aim to compare performance of selected models.

## Figures and Tables

**Table 1:** A summary of recent road damage detection systems for one-class detection.

References	Year	Model	Image Resolution	Inference Speed	Precision	AP@ [0.5:0.95]	AP@0.5
Maeda et al. [38]	2018	SSD using Inception V2	300 × 300	16 FPS	67%	–	–
		SSD MobileNet	300 × 300	33 FPS	99%	–	–
Pena-Caballero et al. [11]	2020	SSD300 MobileNetV2	300 × 300	–	–	–	45.10%
		Yolo v2	–	–	–	–	90.00%
		Yolo v3	–	–	–	–	98.82%
Chen et al. [13]	2020	LACNN	–	49 FPS	95.2%	–	–
Ahmed [10]	2021	YoloR-W6	1774 × 2365	31 FPS	–	44.6%	–
		Faster R-CNN: MVGG16	1774 × 2365	21 FPS	81.4%	45.4%	–
		Yolo v5 (Ys)	1774 × 2365	111 FPS	76.73%	58.9%	–
		Faster R-CNN: ResNet50	1774 × 2365	10 FPS	91.9%	64.12%	–
Lin et al. [39]	2021	Yolo v3	416 × 416	35 FPS	–	–	71%
Park et al. [14]	2021	Yolo v5s	720 × 720	–	82%	–	74.8%
		Yolo v4	720 × 720	–	84%	–	77.7%
		Yolo v4-tiny	720 × 720	–	84%	–	78.7%

**Table 2:** A summary of recent road damage detection systems for multi-class detection.

References	Year	Model	Image Resolution	Inference Speed	mAP@0.5
Pena-Caballero et al. [11]	2020	SSD300 MobileNetV2	300 × 300	–	41.83%
		Yolo v2	–	–	69.58%
		Yolo v3	–	–	97.98%
Lin et al. [39]	2021	MobileNet-Yolo	416 × 416	40 FPS	2.27%
		TF-Yolo	416 × 416	28 FPS	2.66%
		Yolo v3	416 × 416	35 FPS	68.06%
		RetinaNet	416 × 416	30 FPS	73.75%
		Yolo v4	416 × 416	35 FPS	80.08%
		Yolo v4	618 × 618	30 FPS	81.05%
Du & Jiao [15]	2022	Yolo v3-Tiny	640 × 640	167 FPS	59.4%
		Yolo v5S	640 × 640	238 FPS	60.5%
		B-Yolo v5S	640 × 640	278 FPS	62.6%
		BV-Yolo v5S	640 × 640	263 FPS	63.5%

**Table 3:** Available datasets used for road damage detection.

Database	Year	Num. of Images	Num. of Instances	Num. of Classes
MakeML [43]	–	665	–	1
MIIA Pothole Dataset [27]	2015	2459	–	1
Road Damage Dataset [38]	2018	9053	15,435	8
Road Surface Damages [44] (Extended [38])	2019	18,345	45,435	8
Pothole Detection Dataset [42]	2020	1243	–	1
RDD2020 [40]	2020	26,336	>31,000	4
RDD2022 [45]	2022	38,385	55,007	4

**Table 4:** Road damage categories across different datasets.

Database	Categories of Road Damage
Road Damage Dataset [38]	Linear crack, longitudinal wheel mark part Linear crack, longitudinal construction joint part Linear crack, lateral equal interval Linear crack, lateral construction joint part Alligator crack Rutting, bump, pothole, separation White line blur Cross walk blur
RDD2020 [40]	Longitudinal cracks Transverse cracks Alligator cracks Potholes
Pena-Caballero et al. [11]	Longitudinal crack Lateral crack Alligator cracks Potholes Manhole covers Blurred street line Blurred crosswalk

**Table 5:** Statistics of the developed dataset.

Data	Num. of Images	Num. of Instances	Potholes	Manhole Covers
Clear	1052	2128	1896	232
Rain	286	458	383	75
Sunset	201	404	364	40
Evening	250	339	286	53
Night	310	262	220	42

**Table 6:** Performance of Yolo v3 under clear weather conditions.

Model	Image Resolution	Pretrained Weights	Data Augmentation	Precision	Recall	mAP@ 0.5	mAP@ [0.5:0.95]	Inference Speed
Yolo v3	640 × 640	✕	✕	0.434	0.346	0.285	0.092	~35 ms
	640 × 640	✓	✕	0.789	0.512	0.563	0.202	~35 ms
	640 × 640	✕	✓	0.708	0.684	0.681	0.268	~35 ms
	640 × 640	✓	✓	0.713	0.751	0.747	0.314	~35 ms
	1080 × 1080	✓	✓	0.777	0.771	0.771	0.330	~82 ms
Yolo v3-SPP	640 × 640	✓	✓	0.812	0.663	0.711	0.286	~36 ms
	1080 × 1080	✓	✓	0.821	0.700	0.791	0.354	~84 ms

**Table 7:** Performance of the Yolo v3 model (1080 × 1080) under different light and weather conditions.

Data Subset	Class	Image Resolution	Precision	Recall	mAP@0.5	mAP@ [0.5:0.95]
Clear	All	1080 × 1080	0.777	0.771	0.771	0.33
	Potholes		0.726	0.69	0.703	0.262
	Covers		0.828	0.852	0.839	0.398
Rain	All	1080 × 1080	0.613	0.519	0.505	0.199
	Potholes		0.445	0.465	0.396	0.145
	Covers		0.782	0.573	0.614	0.254
Sunset	All	1080 × 1080	0.694	0.496	0.529	0.194
	Potholes		0.537	0.418	0.399	0.133
	Covers		0.852	0.575	0.659	0.256
Evening	All	1080 × 1080	0.742	0.483	0.474	0.182
	Potholes		0.609	0.57	0.518	0.194
	Covers		0.874	0.396	0.429	0.17
Night	All	1080 × 1080	0.36	0.157	0.175	0.062
	Potholes		0.36	0.1	0.145	0.0493
	Covers		0.36	0.214	0.204	0.0746

**Table 8:** Performance comparison of Yolo v3, Yolo v3-SPP and Sparse R-CNN on a clear weather dataset.

Model	Precision	Recall	mAP@0.5	mAP@ [0.5:0.95]	Elapsed Time: Test	Model Size
**Yolo v3**	0.777	0.771	0.771	0.330	24 s	123.7 MB
**Yolo v3-SPP**	0.821	0.700	0.791	0.354	25 s	125.8 MB
**Sparse R-CNN**	–	–	0.726	0.321	31 s	415 MB

## Data Availability

Data supporting reported results can be found at https://doi.org/10.6084/m9.figshare.21214400.v3 (accessed on 16 November 2022).
